# Dynamics of Cations around DNA and Protein as Revealed by ^23^Na Diffusion NMR Spectroscopy

**DOI:** 10.1021/acs.analchem.1c04197

**Published:** 2022-01-26

**Authors:** Binhan Yu, Karina G. Bien, Channing C. Pletka, Junji Iwahara

**Affiliations:** Department of Biochemistry and Molecular Biology, Sealy Center for Structural Biology and Molecular Biophysics, University of Texas Medical Branch, Galveston, Texas 77555-1068 United States

## Abstract

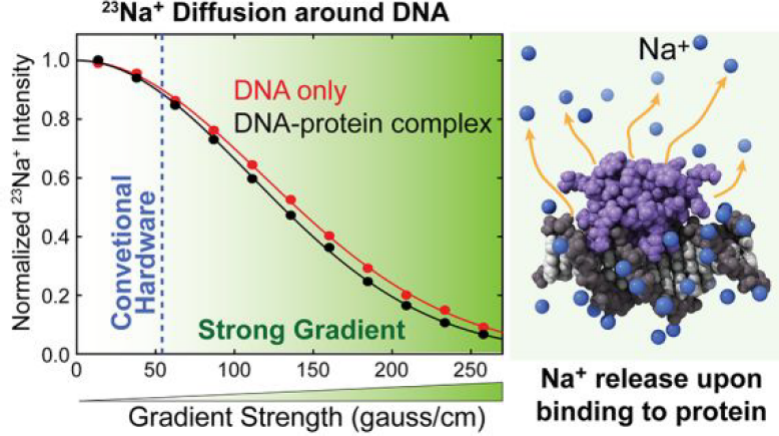

Counterions are vital for the structure and function of biomolecules. However, the behavior of counterions remains elusive due to the difficulty in characterizing mobile ions. Here, we demonstrate that the dynamics of cations around biological macromolecules can be revealed by ^23^Na diffusion nuclear magnetic resonance (NMR) spectroscopy. NMR probe hardware capable of generating strong magnetic field gradients enables ^23^Na NMR-based diffusion measurements for Na^+^ ions in solutions of biological macromolecules and their complexes. The dynamic properties of Na^+^ ions interacting with the macromolecules can be investigated using apparent ^23^Na diffusion coefficients measured under various conditions. Our diffusion data clearly show that Na^+^ ions retain high mobility within the ion atmosphere around DNA. The ^23^Na diffusion NMR method also permits direct observation of the release of Na^+^ ions from nucleic acids upon protein–nucleic acid association. The entropy change due to the ion release can be estimated from the diffusion data.

DNA and RNA are highly negatively charged and electrostatically attract many cations as counterions. Diffusing around nucleic acids, monovalent cations undergo territorial binding^1,2^ (as opposed to site binding) to the macromolecular surfaces and form a zone called the ion atmosphere.^3^ The counterion condensation theory suggests that a large number of counterions are condensed around nucleic acid regardless of the concentration of free cations.^4^ Counterions are vital for the structure and function of nucleic acids.^5,6^ For example, owing to counterions, two negatively charged strands of nucleic acids can be brought in close proximity.^7^ When a protein binds to DNA, counterions are released, which makes a major entropic contribution to the free energy change.^8,9^ Territorial or site bindings of divalent cations also play an important role in RNA structure and dynamics.^10−14^ Thus, it is important to understand how counterions behave around nucleic acids.

In general, however, counterions are difficult to characterize through experiments. Due to the mobile nature, the vast majority of counterions are invisible in crystal structures of nucleic acids and their complexes even at a high resolution. The physical presence of cations condensed around nucleic acids has been demonstrated by other methods, including anomalous small-angle X-ray scattering,^15−18^ atomic emission spectroscopy,^19−22^ and mass spectrometry.^21,23−25^ These methods can detect and quantify cations interacting with DNA or RNA but are not necessarily suited to investigate the characteristics of the bound ions. Despite the wealth of computational studies of ions around DNA and RNA,^1,26−36^ the behavior of counterions within the ion atmosphere remains largely elusive from an experimental perspective.

Nuclear magnetic resonance (NMR) spectroscopy is in principle suitable for characterizing ions around nucleic acids. Over the past 5 decades, NMR has been used to investigate cation–nucleic acid interactions.^37−46^ In 1969, James and Noggle reported a drastic increase in a ^23^Na NMR relaxation rate in the presence of RNA.^47^ Since then, ^23^Na NMR relaxation data have been used to study sodium ion–nucleic acid interactions as well as the relative affinities of other cations that compete with Na^+^ ions for nucleic acids.^38−40,45^^23^Na NMR is convenient because the natural abundance of ^23^Na is 100% and the sensitivity in ^23^Na detection is relatively high. However, because ^23^Na is a quadrupolar nucleus with a spin quantum number of ^3^/_2_, ^23^Na NMR relaxation is rapid due to the quadrupolar mechanism.^48^ Interpretation of ^23^Na NMR relaxation data regarding sodium ion dynamics within the ion atmosphere is difficult due to the lack of experiment-based information on the ^23^Na quadrupolar coupling constant (QCC) for the bound state. The QCC depends on the local electric field around the quadrupole nucleus^48^ and is hard to predict for ^23^Na^+^ ions that are nonspecifically bound to nucleic acids, though QCCs for ^23^Na^+^ ions at particular sites may be predictable.^39^ Despite the long history of ^23^Na NMR, the behavior of Na^+^ ions within the ion atmospheres remains to be elucidated.

In this article, we demonstrate that the dynamic behavior of Na^+^ ions within the ion atmosphere around DNA can be revealed by ^23^Na diffusion NMR spectroscopy. Measuring the diffusion of Na^+^ ions around nucleic acids has been difficult because ^23^Na nuclei exhibit rapid NMR relaxation and a small nuclear gyromagnetic ratio γ (26.45% of the ^1^H γ value), which reduces the extent of spin decoherence via magnetic field gradients. We overcome this obstacle using NMR probe hardware that can generate magnetic field gradients 5 times as strong as the maximum gradients of conventional NMR probe hardware. Unlike previous ^23^Na relaxation-based methods, our current diffusion-based ^23^Na NMR method provides the dynamic properties of Na^+^ ions that are diffusively bound to nucleic acids. We also demonstrate that the ^23^Na diffusion NMR method allows for observation of the release of Na^+^ ions from DNA through competition with other cations or upon the formation of a protein–DNA complex.

## Experimental Section

### DNA and Protein

Individual DNA strands of the 15 base-pair (bp) DNA duplex containing the Antp recognition sequence were prepared as previously described.^49^ The concentrations of the single-stranded DNAs, 5′-dAGAAAGCCATTAGAG-3′ and 5′-dCTCTAATGGCTTTCT-3′, were measured using the UV absorbance at 260 nm along with the extinction coefficients of 1.63 × 10^5^ and 1.30 × 10^5^ M^–1^ cm^–1^, respectively. These extinction coefficients were calculated from the nucleotide sequences using the nearest-neighbor model.^50^ The extinction coefficient at 260 nm for the 15-bp DNA duplex used in this work, 2.17 × 10^5^ M^–1^ cm^–1^, was determined from UV absorbance data for duplex samples prepared through annealing of individual DNA strands at a precisely measured concentration. The ^15^N-labeled fruit fly Antp homeodomain with the C39S mutation was prepared as previously described.^51^ The Antp homeodomain was quantified using the UV absorbance at 280 nm along with the extinction coefficient of 1.55 × 10^4^ M^–1^ cm^–1^ calculated with the Expasy ProtParam tool.^52^

### NMR Samples

**Figure 1:**
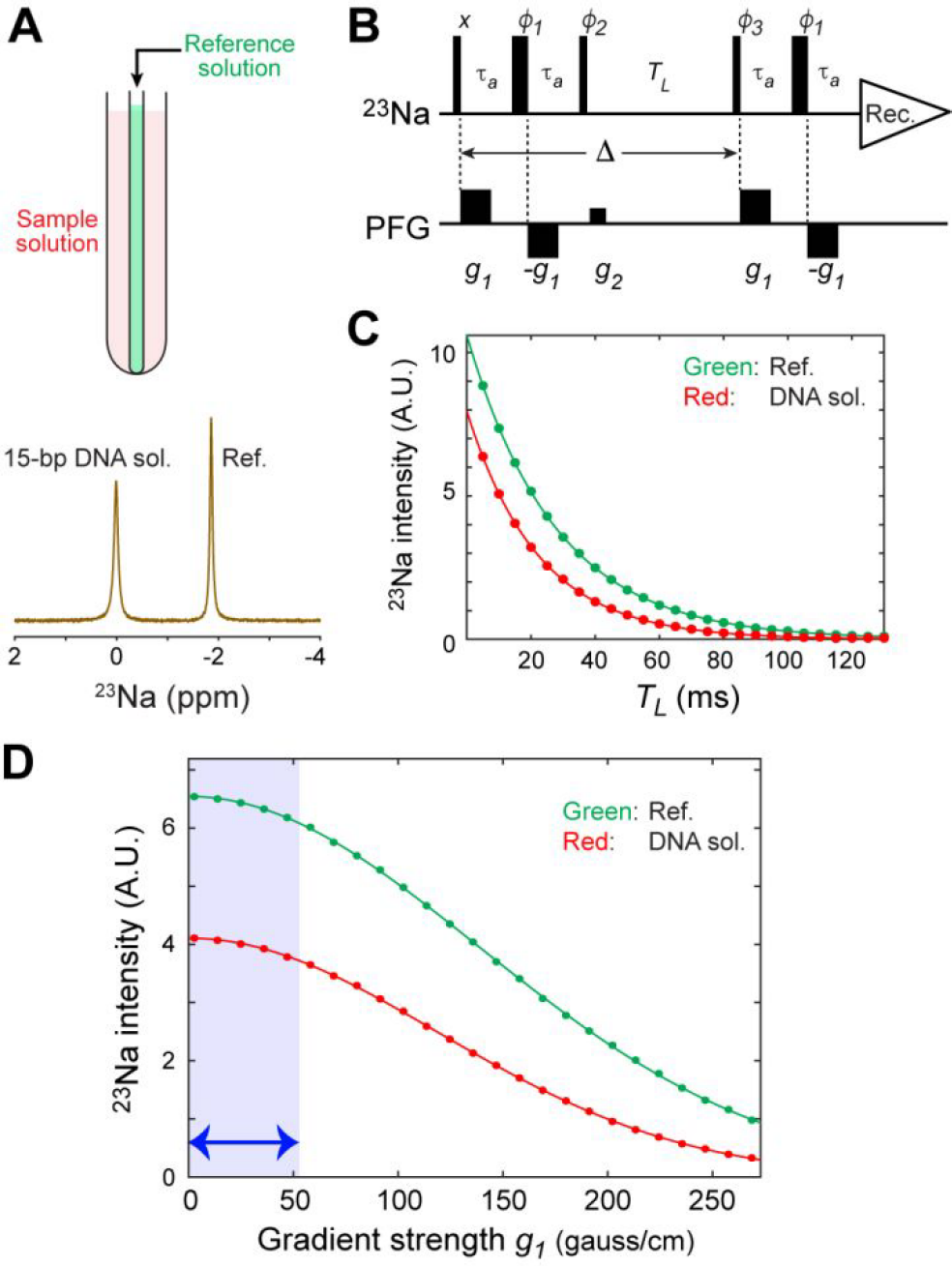
^23^Na diffusion measurements for biomolecular solutions require magnetic field gradients stronger than those available with conventional NMR probe hardware. (A) Coaxial sample configuration used for our ^23^Na diffusion NMR experiments. Also shown is a 1D ^23^Na NMR spectrum recorded for a coaxial sample with the reference solution in the inner tube and a 1.74 mM solution of the 15-bp DNA duplex in the outer tube. The reference solution contains 300 mM NaOH, 20% sulfuric acid, and 80% D_2_O and was designed to provide an isolated ^23^Na signal as a control. (B) The BPP-LED pulse sequence for the ^23^Na diffusion measurements. For ^23^Na pulses, the thin and bold bars represent 90° and 180° pulses, respectively. Phase cycles: *ϕ*_1_ = [4*x*, 4(−*x*)], *ϕ*_2_ = [8*x*, 8(−*x*)], *ϕ*_3_ = [*x*, *y*, −*x*, −*y*], and receiver = [2(*x*, −*y*, −*x*, *y*), 2(−*x*, *y*, *x*, −*y*)]. (C) Signal intensity measured with various values of the delay *T*_L_. The solid lines are the best-fit curves obtained through fitting to a monoexponential function. (D) The ^23^Na signal intensity measured for the aforementioned coaxial sample using the shown pulse sequence at various *g*_1_ field gradients between 0 and 265 G/cm. The delay Δ was 20 ms and each *g*_1_ was 1 ms. The range of field gradients available with a conventional NMR probe hardware (i.e., 55 G/cm) is indicated in blue.

The NMR samples of the 15-bp DNA duplex and the Antp homeodomain (^15^N) were equilibrated with a sodium succinate buffer (pH 5.8) containing 20 mM Na^+^ and 11.3 mM succinate. This buffer, which we hereafter refer to as buffer SS, was prepared through titration of a succinic acid solution into a NaOH solution, lowering the pH to 5.8. This pH was chosen for fair comparison with our previous data on ^15^NH_4_^+^ ions.^44^ The relatively low concentration (20 mM) of Na^+^ ions was chosen so that the ion condensation around the DNA makes a significant impact on the apparent ^23^Na diffusion coefficients for the DNA solutions. The buffer equilibration for the NMR samples was conducted using Amicon Ultra-4 centrifugal filters (molecular weight cutoff at 3 kDa; Millipore) with an overall dilution factor >10 000. A 380 μL solution of each sample was transferred into an outer tube of a 5 mm coaxial NMR tube. Macromolecular concentrations ranged from 0.16 to 1.74 mM. A 110 μL solution containing 300 mM NaOH, 80% (v/v) D_2_O, and 20% (v/v) sulfuric acid, which we refer to as the reference solution, was sealed in a Norell coaxial stem insert (diameter, 2 mm), as depicted in Figure 1A. The reference solution provides a reference ^23^Na signal for Na^+^ quantification (see below) and as well as a ^2^H signal for NMR lock. Another advantage of the use of coaxial tubes is that convection, which may adversely affect diffusion measurements, is suppressed due to the annular geometry.^53^

### NMR Experiments

NMR experiments were conducted at 25 °C with a Bruker Avance III spectrometer operated at a magnetic field of 17.6 T, where the ^1^H and ^23^Na frequencies are 750 and 198 MHz, respectively. All ^23^Na NMR data were recorded using a Bruker DiffBB diffusion broad-band observe probe together with a standard gradient amplifier that produces currents up to 10 A for the gradient coil. This NMR probe hardware can generate magnetic field gradients up to 270 G/cm, which is 5 times as strong as the maximum field gradients of typical probe hardware. ^23^Na chemical shifts were referenced to the ^23^Na signal from the reference solution in the coaxial inner tube (−1.857 ppm with respect to 0.1 M NaCl in D_2_O). ^23^Na pulses at a radio frequency (RF) strength of 14.6 kHz were used to record NMR data. ^23^Na diffusion NMR data were recorded using the bipolar pulsed-gradient pair longitudinal eddy-current delay (BPP-LED) pulse sequence (Figure 1B) along with 11–25 gradient strengths ranging from 2 to 265 G/cm for dephasing and rephasing. The pulsed field gradient strengths were calibrated with reference to the self-diffusion coefficient (1.63 × 10^–5^ cm^2^ s^–1^) of liquid *N*,*N*-dimethylformamide at 25 °C.^54^ Each diffusion coefficient *D* was determined from BPP-LED data through nonlinear least-squares fitting to experimental signal integral data using Bruker Topspin 3.2 software. The relationship between the signal intensity *I* and the pulse field gradient *g* is^55^
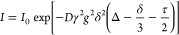
1in which *D* is the diffusion coefficient, γ is the nuclear gyromagnetic ratio, *g* is the magnetic field gradient strength, δ is the total length of a pair of bipolar gradients, Δ is the time between the beginning points of two spin echo periods, and τ is the time between two gradients in each spin echo. NMR experiments for each sample were replicated three times. Error bars in figures and uncertainties in measured values represent the standard error of the mean.

### Quantification of Na^+^ Ions in Macromolecular Solutions

Due to counterions accumulating around charged macromolecules, the total concentration of Na^+^ ions in a macromolecular solution can differ from the concentration of Na^+^ ions in the buffer alone. The total concentration of Na^+^ ions in each macromolecular solution ([Na^+^]_total_) was determined from the ratio of the integral of the ^23^Na NMR signal from the macromolecular solution to the integral of that from the reference solution (*r* = *I*/*I*_ref_). Using buffer SS alone in the outer tube, the integral ratio for 20 mM Na^+^ (*r*_buffer_) was obtained as the standard for the quantification. The Na^+^ concentration in each sample was calculated as 20*r*_sample_/*r*_buffer_ mM. For each measurement, an identical coaxial stem insert containing the reference solution was used to avoid deviations of the reference intensity caused by variations in thickness of the coaxial stem insert glass wall. Manning’s counterion condensation theory suggests that the number of counterions that accumulate around each DNA molecule is independent of the concentrations of free ions.^4^ The dependence of [Na^+^]_total_ on the macromolecular concentration was analyzed using
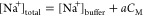
2[Na^+^]_buffer_ is the concentration of Na^+^ ions in the buffer (i.e., 20 mM), *C*_M_ is the macromolecular concentration, and *a* is the number of Na^+^ ions within the ion atmosphere around the macromolecule. The parameter *a* was determined from the *C*_M_-dependent [Na^+^]_total_ data.

### Diffusion-Based Analysis of Na^+^ Ions within the Ion Atmosphere

The apparent diffusion coefficient (*D*_app_) of Na^+^ ions was measured for the solutions of the 15-bp DNA duplex at eight different concentrations (0.21, 0.47, 0.66, 0.88, 1.09, 1.44, 1.52, and 1.74 mM). To analyze the diffusional properties of Na^+^ ions within the ion atmosphere around DNA, the following equation for fast exchange systems^44,56^ was used to analyze the *D*_app_ data:
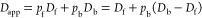
3where *p* represents a population and *D* is an intrinsic diffusion coefficient; the annotations f and b are for Na^+^ ions in the free state and those in the territorially bound state (i.e., within the ion atmosphere), respectively. On the basis of eq 2, the population *p*_b_ for Na^+^ ions in DNA solutions where sodium ions are the only cations is given by
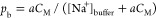
4

*D*_f_ was directly measured for buffer SS. The parameter *a* was experimentally determined, as described above. The diffusion coefficient *D*_b_ for Na^+^ ions within the ion atmosphere was determined from *C*_M_-dependent *D*_app_ data through nonlinear least-squares fitting with eqs 3 and 4. The diffusion coefficient *D*_b_ was the only fitting parameter in this calculation. The fitting was performed using MATLAB (MathWorks).

### Ionic Competition between Na^+^ and K^+^ Ions for DNA

To investigate ionic competition between Na^+^ and potassium (K^+^) ions for the ion atmosphere around DNA, KCl was added to a solution of 1.74 mM 15-bp DNA duplex equilibrated with buffer SS. The apparent diffusion coefficient of Na^+^ ions was measured at various concentrations of KCl. The [KCl] dependence data of the Na^+^ diffusion coefficient was analyzed using a competition parameter (*Q*) defined as follows:^38^
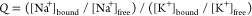
5The competition parameter *Q* is an equilibrium quotient that remains constant throughout the experiment. The apparent Na^+^ diffusion coefficient *D*_app_ in the presence of KCl is given by^44^
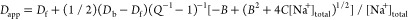
6where *C* = *n*_c_[DNA](*Q*^–1^ – 1), *B* = [Na^+^]_total_ + *Q*^–1^[KCl] – *C*, and *n*_c_ represents a total number of monovalent cations in the ion atmosphere. The parameter *Q* was determined from *D*_app_ data at various concentrations of KCl through nonlinear least-squares fitting with MATLAB. In the fitting calculation, the values of *D*_f_, *D*_b_, and [Na^+^]_total_ were set to those determined from the DNA concentration dependence data (see above) and the parameter *n*_c_ was set equal to the parameter *a* (eq 2) determined for the system where Na^+^ ions are the only cations.

### Analysis of Protein-Induced Na^+^ Release from DNA

The release of Na^+^ ions from DNA upon formation of a protein–DNA association was investigated through ^23^Na diffusion experiments for two samples. One sample was a solution containing 1.52 mM 15-bp DNA duplex. The other sample was a solution of 1.52 mM 15-bp DNA and 1.10 mM Antp homeodomain. These samples were prepared using a 3.04 mM DNA solution and a 2.20 mM protein solution, both of which were equilibrated with buffer SS. The two NMR samples were prepared by mixing 190 μL of the DNA solution with 190 μL of either the protein solution or buffer SS. For each sample, the apparent diffusion coefficients of the Na^+^ ions were measured using the ^23^Na BPP-LED pulse sequence. For the protein–DNA sample, the formation of the Antp homeodomain–DNA complex was confirmed by recording a ^1^H–^15^N heteronuclear single-quantum coherence (HSQC) spectrum. The number of Na^+^ ions released upon protein–DNA association (*n*_R_) was estimated from the diffusion data using the following equation:^44^
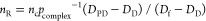
7*D*_PD_ and *D*_D_ are the apparent diffusion coefficients for the protein–DNA and DNA samples, respectively, and *p*_complex_ is the fraction of the 15-bp DNA duplex bound to the protein.

## Results and Discussion

Through ^23^Na diffusion NMR experiments using strong field gradients, we investigated the dynamic behavior of Na^+^ ions around the 15-bp DNA duplex and the Antp homeodomain. This protein recognizes the TAATGG sequence within double-stranded DNA and binds to the 15-bp DNA duplex with a dissociation constant (*K*_d_) of 10^–9^–10^–8^ M under physiological conditions.^51^ This macromolecular system is well-suited for our current investigations on Na^+^ ions, particularly because the diffusional properties and spatial distribution of other ions around these macromolecules were examined previously.^44,57,58^ In our current NMR study, two ^23^Na signals were observed in each experiment: one from a 380 μL macromolecular solution in the outer tube, and the other from a 110 μL reference solution containing 300 mM NaOH, 20% (v/v) sulfuric acid, and 80% (v/v) D_2_O in the inner tube (Figure 1A). The reference solution was designed to yield a distinct, well-isolated ^23^Na resonance that serves as a control in our analysis of Na^+^ ions in the macromolecular solution. Despite the presence of Na^+^ ions in the free state and in the bound state, each DNA solution in the outer tube exhibited a single ^23^Na signal, indicating that Na^+^ ions in the free state and those in the bound state undergo fast exchange, as previously reported.^38−40,45^

### Effectiveness of Strong Magnetic Field Gradients

Using the ^23^Na BPP-LED pulse sequence^55^ shown in Figure 1B, we measured the diffusion of Na^+^ ions. ^23^Na NMR relaxation is rapid due to the quadrupole relaxation mechanism.^48^ Na^+^ ions in DNA solutions exhibit particularly rapid relaxation. A relatively small nuclear gyromagnetic ratio and the rapid decay of ^23^Na NMR signals make it difficult to measure ^23^Na diffusion using typical broad-band NMR probe hardware. Figure 1C shows ^23^Na signal intensities measured using various lengths of the delay *T*_L_ in a ^23^Na BPP-LED experiment for Na^+^ ions in a 1.74 mM 15-bp DNA solution and Na^+^ ions in the reference solution. The data clearly show that rapid decays through relaxation severely limit the practical range of the delay *T*_L_ for measuring diffusion of Na^+^ ions in DNA solutions. Therefore, sizable dephasing effects essential for NMR-based diffusion measurements should be achieved using strong magnetic field gradients.

A typical range of magnetic field gradients of conventional broad-band NMR probe hardware (up to ∼55 G/cm) is insufficient to precisely measure the diffusion of ^23^Na^+^ ions in DNA solutions. As shown in Figure 1D, using magnetic field gradients up to 265 G/cm, we were able to achieve >85% attenuation of ^23^Na signals through diffusion in the ^23^Na BPP-LED experiment with *T*_L_ = 20 ms and varied strengths of 1 ms pulsed field gradients (PFGs) *g*_1_. With a conventional broad-band NMR probe, the range of the diffusion-induced attenuation under the same condition would be only <10%, as indicated by the blue region in Figure 1D. The NMR probe hardware capable of generating strong field gradients allowed us to precisely measure the diffusion of Na^+^ ions in DNA and protein solutions under various conditions.

### Condensation of Na^+^ Ions around DNA

**Figure 2:**
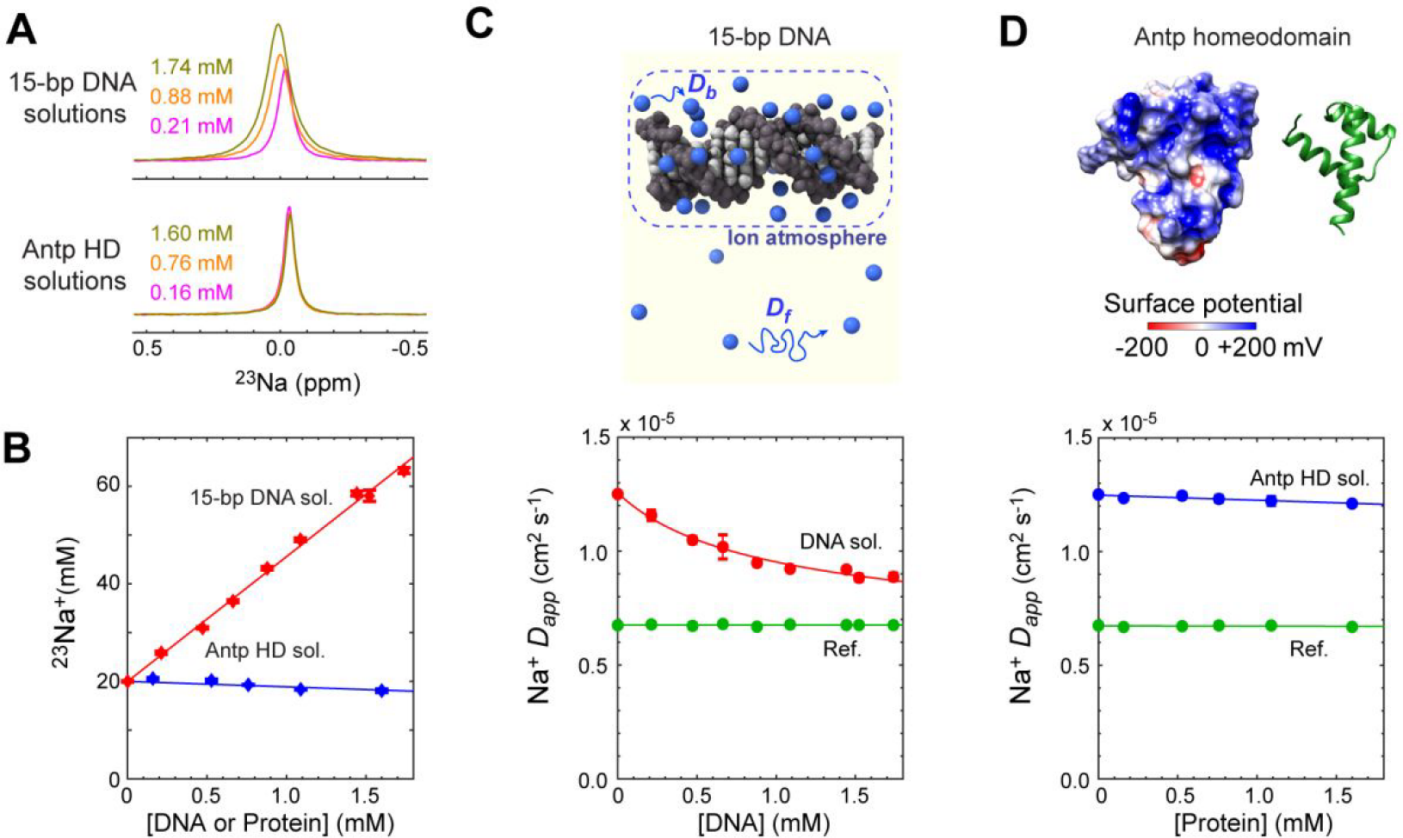
^23^N diffusion data indicating the behavior of Na^+^ ions around the 15-bp DNA duplex and the Antp homeodomain (HD). (A) ^23^Na NMR signals observed for the DNA solutions and the protein solutions. (B) Total Na^+^ concentrations measured for the DNA solutions and the protein solutions equilibrated with the sodium succinate buffer (pH 5.8) containing 20 mM Na^+^ ions as the only cations. The signal integrals of the ^23^Na signals from the samples in the outer tube and from the reference solution in the inner tube (see Figure 1A) were used to measure the total Na^+^ concentration in each sample. (C) The apparent diffusion coefficients of Na^+^ ions in the solutions of the 15-bp DNA duplex at various concentrations. The solid red line represents the best-fit curve obtained through nonlinear least-squares fitting using eqs 3 and 4, which determined the diffusion coefficient of Na^+^ ions within the ion atmosphere (*D*_b_). See Table I for the values of the diffusion coefficients *D*_f_ and *D*_b_. (D) The apparent diffusion coefficients of Na^+^ ions in the solutions of the Antp homeodomain at various concentrations. In panels C and D, the diffusion data on Na^+^ ions in the reference solution measured in each experiment are shown in green.

We recorded ^23^Na NMR spectra for Na^+^ ions in solutions of the 15-bp DNA duplex and in solutions of the Antp homeodomain at various concentrations. As shown in Figure 2A, the chemical shift, line shape, and intensity of the NMR signal from Na^+^ ions in the DNA solutions depended on the DNA concentration. In contrast, the NMR signal from Na^+^ ions in the Antp homeodomain solutions exhibited very little dependence on the protein concentration. Since counterions condensed around macromolecules do not pass through the centrifugal filter membrane in the buffer equilibration process,^19^ the total Na^+^ concentrations in the DNA solutions are higher than in the buffer. Using the integrals of the ^23^Na NMR signals from the outer and inner tubes, we measured the total Na^+^ concentration in each sample (Figure 2B). The total Na^+^ concentration was linearly dependent on the DNA concentration. The slope of the linear dependence (i.e., the parameter *a* in eq 2) represents the number of Na^+^ ions around each DNA molecule. Because Na^+^ ions are the only cations in the current case, this number corresponds to the ion excess, Δ*N*_cation_, which is the difference between the number of cations in the ion atmosphere and the number of cations in the same volume outside the ion atmosphere.^59^ From the NMR data, we determined the ion excess to be 25.5 ± 1.7. This value was 18% larger than the prediction for the same DNA (Δ*N*_cation_ = 21.6)^57^ from the nonlinear Poisson–Boltzmann equation based electrostatic potentials calculated by the APBS software.^60^ Using buffer equilibration and atomic emission spectroscopy, Bai et al. also found that the number of cations condensed around DNA was systematically larger than predictions from the Poisson–Boltzmann theory.^19^ Our NMR data show that Na^+^ ions are condensed around the negatively charged DNA duplex (overall charge, −28*e*) but not around the positively charged protein (overall charge, +14*e* at pH 5.8).

### Na^+^ Diffusion around DNA

We measured the diffusion coefficients of ^23^Na^+^ ions at various concentrations of the 15-bp DNA duplex. In each diffusion experiment, diffusion coefficients were determined for Na^+^ ions in the outer tube and those in the inner tube (i.e., the reference solution). The diffusion coefficient of Na^+^ ions in the reference solution (0.675 × 10^–5^ cm^2^ s^–1^) was smaller than in the buffer (1.251 × 10^–5^ cm^2^ s^–1^), which can be attributed to a higher viscosity of the reference solution containing 20% sulfuric acid (1.61 mPa s)^61^ than that of water (0.89 mPa s)^62^ at 25 °C. The diffusion coefficient of Na^+^ ions in the DNA solutions was found to nonlinearly depend on the DNA concentration (Figure 2C), while the diffusion coefficient of Na^+^ ions in the reference solution remained constant. As described in our previous study on NH_4_^+^ ions,^44^ the DNA concentration dependence of the apparent diffusion coefficients can be explained using a model involving the diffusion coefficient of free Na^+^ ions (*D*_f_) and the diffusion coefficient of Na^+^ ions within the ion atmosphere around DNA (*D*_b_). The red solid line in Figure 2C is the best-fit curve obtained with the model represented by eqs 3 and 4. Although the curve fitting was performed through optimization of a single parameter (i.e., *D*_b_), excellent fitting was obtained, supporting the appropriateness of the model. The diffusion coefficient *D*_b_ was determined to be (0.71 ± 0.02) × 10^–5^ cm^2^ s^–1^.

Table I compares the diffusional properties of Na^+^ and NH_4_^+^ ions around DNA from our current and previous^44^ studies. The *D*_f_ coefficients are consistent with the known diffusion coefficients of free Na^+^ and NH_4_^+^ ions in water at 25 °C.^62^ It may seem counterintuitive that Na^+^ diffusion is slower than NH_4_^+^ diffusion although the ionic radius of Na^+^ is smaller. This can be explained by stronger interactions of sodium with water molecules due to a higher charge density of Na^+^. If all counterions undergo site binding, the *D*_b_ coefficients should be identical to the diffusion coefficient of the 15-bp DNA (0.10 × 10^–5^ cm^2^ s^–1^).^44^ However, the actual *D*_b_ coefficients were 6–10-fold larger than this expectation. The *D*_b_ data suggest that Na^+^ and NH_4_^+^ ions remain highly mobile in the ion atmosphere and only moderately lose their mobility through territorial binding to DNA.

**Table I:** Comparison of the Diffusional Properties of Na^+^ and NH_4_^+^ Ions around DNA

cation	Na^+^	NH_4_^+^^a^
*D*_f_ (cm^2^ s^–1^)^b^	(1.251 ± 0.003) × 10^–5^	(1.83 ± 0.02) × 10^–5^
*D*_b_ (cm^2^ s^–1^)^c^	(0.71 ± 0.02) × 10^–5^	(1.08 ± 0.06) × 10^–5^
*D*_b_/*D*_f_	0.57 ± 0.02	0.59 ± 0.03
Δ*S*_release_ (eu per ion)^d^	1.13 ± 0.06	1.05 ± 0.11

a Data from the ^15^N NMR study by Pletka et al (ref (44)).

b The diffusion coefficient of cations in the free state.

c The diffusion coefficient of cations within the ion atmosphere around DNA.

d Entropic change per ion due to the release from DNA. Estimated from *D*_b_/*D*_f_ along with the equation of Seki and Bagchi (ref (64)).

Interestingly, the ratio *D*_b_/*D*_f_ was virtually identical for Na^+^ and NH_4_^+^ ions despite their different diffusional properties. This ratio might be independent of charge density, though further studies on other monovalent cations are obviously required to examine this possibility. Manning’s theory on ionic diffusion in the presence of polyelectrolytes seems to support this possibility.^63^ On the basis of Seki–Bagchi theory on the relationship between entropy and diffusion,^64^ the entropy change upon the release of a cation from the ion atmosphere (Δ*S*_release_) is estimated to be −*k*_B_ ln(*D*_b_/*D*_f_), where *k*_B_ is the Boltzmann constant. The similar *D*_b_/*D*_f_ ratios for Na^+^ and NH_4_^+^ ions suggest that the release of these monovalent cations makes a similar entropic contribution per ion to the free energy change upon DNA–protein association.

### Na^+^ Diffusion around Protein

We also measured the diffusion coefficient of Na^+^ ions in the Antp homeodomain solutions (Figure 2D). This protein contains 20 basic side chains (12 arginine, 2 histidine, and 6 lysine residues) and 6 acidic side chains (6 glutamate residues). The diffusion coefficient of the Na^+^ ions remained almost constant at relatively low concentrations of the protein. When the protein concentration exceeded 1 mM, a slight decrease in the Na^+^ diffusion coefficient became evident, presumably due to the macromolecular crowding effect.^57^ The magnitude of the decrease was far smaller than that observed for the DNA solutions. Interestingly, our ^1^H diffusion experiments for succinate in the same solutions showed an opposite trend: strong dependence on the protein concentration and weak dependence on the DNA concentration (Figure S1). This is consistent with our recent study on acetate (OAc^–^) ions.^57^ Since the vast majority of succinate molecules are either monovalent (40%) or divalent (58%) anions at pH 5.8, the positively charged Antp homeodomain attracts succinate anions, causing a decrease in their apparent diffusion coefficient. These diffusion data clearly demonstrate that diffusion NMR spectroscopy is powerful for investigating ions that are electrostatically interacting with biological macromolecules.

### Na^+^ Release from DNA through Competition with K^+^ Ions

**Figure 3:**
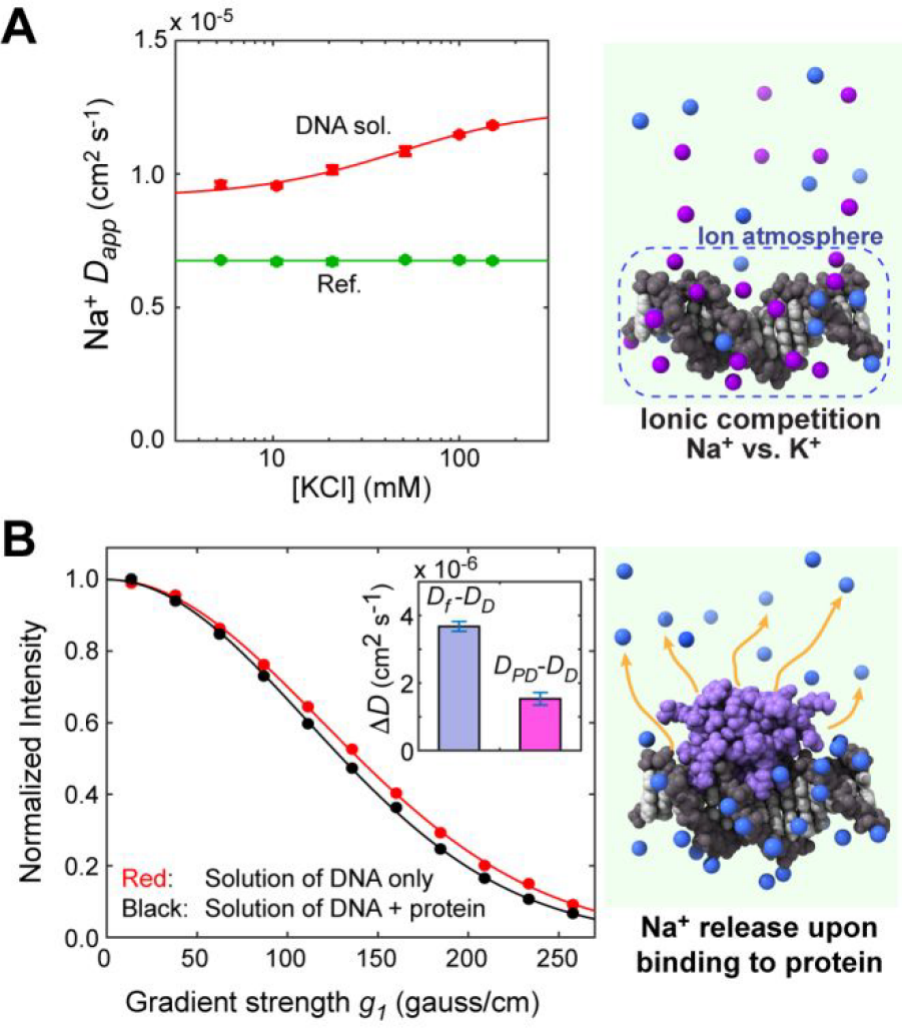
Release of Na^+^ ions from DNA observed by ^23^Na diffusion NMR spectroscopy. (A) The change in the apparent ^23^Na diffusion coefficient due to the release of Na^+^ ions from the 15-bp DNA duplex through competition with K^+^ ions for the ion atmosphere. KCl was added to the solution of 1.74 mM DNA equilibrated with buffer SS. The release of Na^+^ ions from the 15-bp DNA duplex upon association with the protein causes an increase in the observed diffusion coefficient of Na^+^ ions. (B) ^23^Na diffusion NMR based observation of the Na^+^ release upon protein–DNA association.

Through ^23^Na diffusion experiments, we also investigated the competition between Na^+^ and K^+^ ions for the ion atmosphere around DNA. In this experiment, KCl was added to a solution of the 15-bp DNA duplex equilibrated with buffer SS. The addition of K^+^ ions should reduce the population of ^23^Na^+^ ions in the ion atmosphere. In fact, as shown in Figure 3A, faster diffusion of ^23^Na^+^ ions was observed when the K^+^ concentration was increased. This is not due to a change in viscosity because KCl at a concentration in the range of 0.01–0.15 M causes only a very small change in the viscosity at 25 °C by 0.2% or less.^65^ We measured apparent diffusion coefficients of Na^+^ ions at various concentrations of KCl and fit the data using the model represented by eq 6. This model assumes that DNA exhibits different binding preferences for Na^+^ and K^+^ ions. The competition parameter *Q* defined by eq 5 represents a relative preference. As seen in Figure 3A, the fitting was excellent although *Q* was the only optimized parameter. The fitting calculation yielded *Q* = 0.58 ± 0.09, suggesting that DNA has a stronger preference for K^+^ ions than for Na^+^ ions. Since the competition parameter *Q* (eq 5) was determined to be 1.89 for NH_4_^+^ versus K^+^ ions (which was measured as *Q*^–1^ = 0.53 in ref (44)), the preference order is NH_4_^+^ > K^+^ > Na^+^ for the territorial binding of cations to DNA. The same preference order was reported by Bleam et al. as well.^38^ Some computational studies showed that Na^+^ and K^+^ ions exhibited different trends in spatial distributions around nucleic acid surfaces: higher occupancies of Na^+^ ions at backbone phosphates and higher occupancies of K^+^ ions at nucleotide bases.^31,66,67^ Our NMR diffusion data clearly show that K^+^ ions can effectively expel Na^+^ ions from the ion atmosphere around DNA despite the predicted difference in preferential interaction sites of K^+^ and Na^+^ ions.

### Release of Na^+^ Ions upon Protein–DNA Association

When proteins bind to DNA, charge neutralization via ion pairs of DNA phosphates and protein basic side chains causes the release of counterions from DNA.^6^ This release has been considered to make a major contribution to the binding free energy.^8^ In the current study, taking advantage of ^23^Na diffusion NMR spectroscopy with strong field gradients, we investigated the release of Na^+^ ions from DNA upon protein–DNA association. The diffusion-based approach is well-suited for this purpose because the counterion release causes an increase in the populations of free ions which undergo faster diffusion, as we previously demonstrated for NH_4_^+^ and OAc^–^ ions.^44,57^

We performed the ^23^Na diffusion experiments for a solution of 1.52 mM 15-bp DNA and 1.10 mM Antp homeodomain and a solution of 1.52 mM 15-bp DNA alone. Due to the high affinity of the Antp homeodomain for this DNA duplex,^51^ virtually all proteins bind to DNA in this protein–DNA solution. As shown in Figure 3B, the formation of the complex between the Antp homeodomain and the 15-bp DNA duplex caused faster Na^+^ diffusion than in the solution of DNA alone at the same concentration. The apparent diffusion coefficients were determined to be (1.036 ± 0.012) × 10^–5^ cm^2^ s^–1^ for Na^+^ ions in the protein–DNA solution (*D*_PD_) and (0.883 ± 0.014) × 10^–5^ cm^2^ s^–1^ for Na^+^ ions in the solution of DNA alone (*D*_D_). The observed change *D*_PD_–*D*_D_ corresponds to a significant portion of the maximum difference *D*_f_ – *D*_D_ (see the inset in Figure 3B), suggesting that some Na^+^ ions are released from DNA upon the formation of the complex. Using eq 7, the number of the released Na^+^ ions (*n*_R_) was estimated to be 14.7 ± 1.7 for the Antp homeodomain–DNA complex. This result from our direct observation of the ion release is significantly larger than the previous indirect estimate from the salt concentration dependence of the binding equilibrium constant (6.9 ± 0.3).^68^ Regarding this discrepancy, we should point out that the high mobility of counterions within the ion atmosphere substantially reduces the entropic increase per ion.

### ^23^Na Relaxation Rates from Line-Shape Analysis

**Figure 4:**
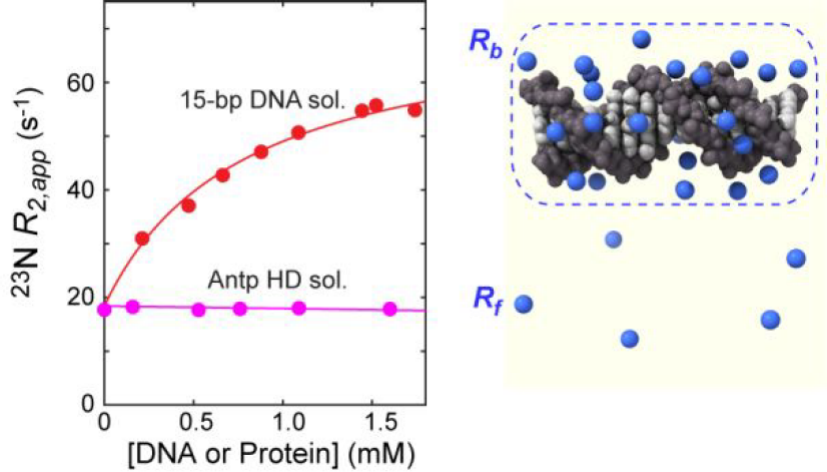
^23^Na NMR line-shape-based analysis of interactions between Na^+^ ions and DNA. The apparent ^23^Na transverse relaxation rate *R*_2,app_ was determined through NMR line-shape fitting for the ^23^Na BPP-LED data (see also Figure S2). The relaxation rate for Na^+^ ions within the ion atmosphere around DNA (*R*_b_) was determined to be 73 ± 2 s^–1^.

Conventional ^23^Na line-shape analysis is also feasible using the data obtained for ^23^Na diffusion NMR spectroscopy. Adverse effects of the eddy currents induced by altering magnetic fields are effectively eliminated in the ^23^Na BPP-LED pulse sequence (Figure 1B),^69^ making it possible to quantitatively analyze ^23^Na NMR line shapes recorded in diffusion experiments. As described in the Supporting Information, the apparent ^23^Na *R*_2_ relaxation rate (*R*_2,app_) for Na^+^ ions was determined through the NMR line-shape fitting to the ^23^Na BPP-LED data for each macromolecular solution (Figure 4; see also Figure S2). The ^23^Na *R*_2_ relaxation rate for Na^+^ ions in the free state (*R*_f_) was determined to be 17.3 ± 0.2 s^–1^ from the data on the sample of the buffer alone. The *R*_2,app_ rates for Na^+^ ions in the 15-bp DNA solutions were considerably larger, ranging from 30.5 to 54.4 s^–1^, in a manner dependent on the DNA concentration. From these *R*_2,app_ data, the *R*_2_ relaxation rate for Na^+^ ions within the ion atmosphere (*R*_b_) was determined to be 73 ± 2 s^–1^, as described in the Supporting Information. The red solid line in Figure 4 is the best-fit curve. Although the *R*_b_ rate was the only parameter optimized in the curve fitting, an excellent fitting was achieved. This result with the simple model for *R*_2,app_ rates without the exchange contribution term (*R*_ex_) suggests that the residence time of Na^+^ ions in the ion atmosphere is far shorter than the inverse of the chemical shift difference between the free and bound states.^39^ However, unlike the case with the *D*_f_ and *D*_b_ coefficients described above, the *R*_f_ and *R*_b_ rates do not provide direct insight into the physical properties of Na^+^ ions inside and outside of the ion atmosphere because the quadrupole relaxation depends on not only the rotational correlation time but also local electric fields at various locations.

^23^Na *R*_2,app_ data can provide some information about the ion release events. In fact, our diffusion NMR data also showed a decrease in the *R*_2,app_ rate when KCl was added in the ionic competition experiment (Figure S3). As pioneered by Bleam et al., the ^23^Na NMR line-shape data can provide quantitative information on the competition between Na^+^ and other cations.^38^ Our diffusion NMR data also showed a decrease in the *R*_2,app_ rate upon protein–DNA association, reflecting an increase in the population of Na^+^ ions in the free state. However, when a model corresponding to eq 6 with the diffusion coefficients replaced with the relaxation rates was used to fit the *R*_2,app_ data at varied concentrations of KCl, systematic deviations from the experimental data were observed (Figure S3). This might suggest that ^23^Na transverse relaxation rates *R*_b_ and *R*_f_ may depend on the KCl concentration, possibly due to transient interactions with Cl^–^ ions that affect local electric fields. We should also point out that it is impractical to estimate *n*_R_ from the *R*_2,app_ data because the formation of the protein–DNA complex can change the effective ^23^Na QCC and the rotational correlation time relevant to the *R*_b_ rate for Na^+^ ions within the ion atmosphere. Thus, to characterize the sodium dynamics, diffusion data are better suited than relaxation data.

## Conclusions

We have demonstrated that ^23^Na diffusion NMR spectroscopy using strong magnetic field gradients enables detailed characterization of Na^+^ ions condensed around DNA and protein molecules. Although ^23^Na diffusion spectroscopy using strong magnetic field gradients was previously applied to investigations of other materials,^70,71^ its applications to DNA and protein are unprecedented and provide unique insight into the behavior of cations around the biomolecules. Our data show that Na^+^ ions within the ion atmosphere retain high mobility while they are territorially bound to DNA. ^23^Na diffusion NMR spectroscopy also allows us to observe the release of Na^+^ ions from DNA through competition with other ions or a protein that binds to DNA. When counterions are released from DNA upon protein–DNA association, their high mobility within the ion atmosphere seems to considerably reduce the entropic increase per ion. Although our current study used a relatively small macromolecular system, the approach is not limited by macromolecular size and will be able to reveal sodium ion dynamics for various nucleic acids and proteins. In principle, the same diffusion-based approach would be applicable to other NMR-detectable ions, though low-γ quadrupole nuclei such as ^35^Cl^–^ may require even stronger field gradients. Applications of the current approach to various systems will facilitate experiment-based examination of theoretical and computational models on electrostatic interactions and help advance our understanding of structure and function of biomolecules and their complexes.

## Supporting Information Available

Diffusion data for succinate ions in protein and DNA solutions and details of the line-shape analysis for ^23^N diffusion NMR data (PDF)
