# Supplementary material for: Dynamics of Cations around DNA and Protein as Revealed by 23Na Diffusion NMR Spectroscopy
Source: Anal Chem. 2022 Jan 26;94(5):2444–52. doi: 10.1021/acs.analchem.1c04197 (PMC8829827; doi:10.1021/acs.analchem.1c04197)
Supplement: Supplementary file 1 — ac1c04197_si_001.pdf [file ac1c04197_si_001.pdf]

SUPPLEMENTARY INFORMATION

Dynamics of cations around DNA and protein as revealed by ^{23}Na diffusion NMR spectroscopy

Binhan Yu, Karina G. Bien, Channing C. Pletka, and Junji Iwahara

Department of Biochemistry & Molecular Biology, Sealy Center for Structural Biology & Molecular Biophysics, University of Texas Medical Branch, Galveston, Texas 77555-1068 USA

Table of Content

1. Diffusion data for succinate ions in protein and DNA solutions
2. Details of line shape analysis for ^{23}Na diffusion NMR data

1. Diffusion data for succinate ions in protein and DNA solutions

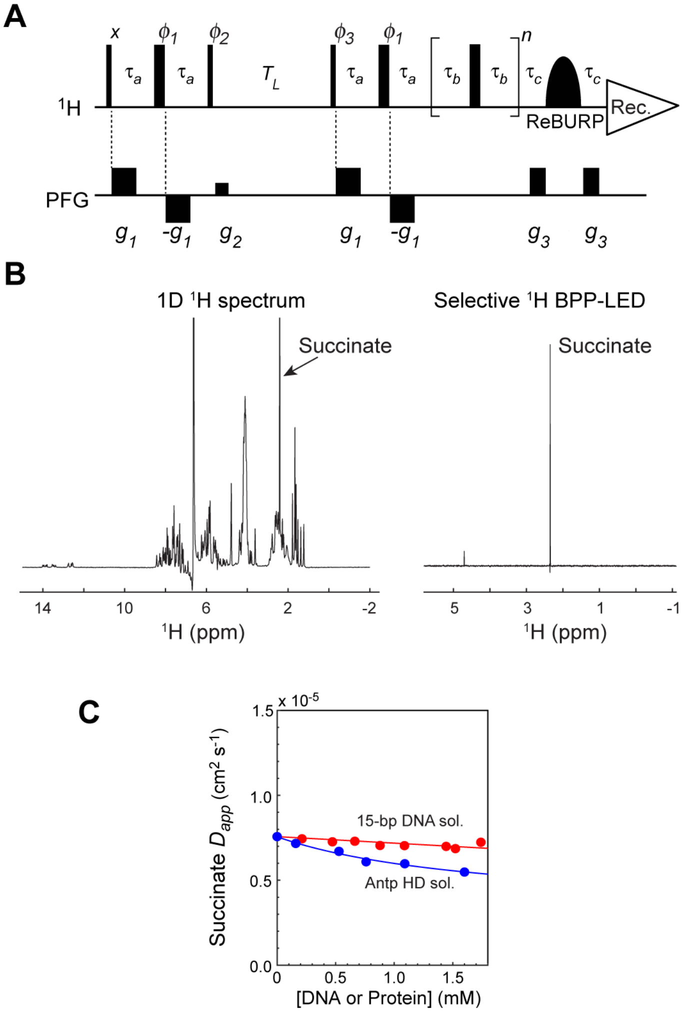

Fig. S1. Diffusion measurements for succinate in the 15-bp DNA solutions and the Antp homeodomain solutions used for Figure 2. (**A**) Selective ^{1}H BPP-LED pulse sequence used for the succinate diffusion measurements. In this pulse sequence, the BPP-LED scheme is followed by a 400 ms relaxation filter and a spin-echo scheme using a 10 ms REBURP refocusing pulse for selective observation of succinate methylene ^{1}H signal at 2.35 ppm. The phase cycles are the same as those indicated in the caption for Figure 1B. Gradient strengths used for succinate diffusion measurement were: 5.4, 10.3, 15.2, 20.1, 24.9, 29.8, 34.7, 39.6, 44.5, 49.3 and 54.2 gauss/cm. (**B**) The relaxation filter and the spin-echo using a shaped pulse effectively eliminated signals from the macromolecular components. (**C**) Apparent diffusion coefficients of succinates in the DNA solutions and in the protein solutions at 25˚C. These diffusion coefficients were measured immediately after ^{23}Na diffusion measurements using the same NMR equipment at the ^{1}H frequency of 750 MHz.

2. Details of line shape analysis for ^{23}Na diffusion NMR data

NMR line-shapes of ^{23}Na signals were analyzed using the ^{23}Na diffusion NMR data. The resonance positions and the transverse relaxation rates were obtained through nonlinear least-squares fitting to the spectra using the sum of the Lorentzian functions:

$$A(\delta) = \sum_i^n \frac{s_i R_{eff,i}}{R_{eff,i}^2 + 4\pi^2(\delta - \delta_i)^2} \qquad \text{[S1].}$$

$A(\delta)$ represents the amplitude; δ, a position in the frequency domain in Hz; $R_{eff,i}$, the effective transverse relaxation rate for the ith signal in s^{-1}; and δ_i, chemical shift of the ith signal in Hz. In the current case, $n = 2$ with a ^{23}Na signal from the macromolecular solution in the outer tube and another ^{23}Na signal from the reference solution in the inner tube. The apparent ^{23}Na transverse relaxation rate $R_{2,app}$ is equal to $R_{eff} - \pi L_B$, where L_B is the line broadening factor L_B (in Hz) of the exponential window function used for processing the time-domain NMR data. Assuming that the exchange between Na^+ ions inside and outside the ion-atmosphere occurs through diffusion very rapidly, the apparent transverse relaxation rate $R_{2,app}$ can be given by:[46]

$$R_{2,app} = p_f R_f + p_b R_b = R_f + p_b(R_b - R_f) \qquad \text{[S2],}$$

in which R_f and R_b represent the intrinsic transverse relaxation rates for Na^+ ions in the free state and Na^+ ions territorially bound to DNA (i.e., within the ion atmosphere), respectively. The relaxation rate R_f was directly measured for Na^+ ions in the buffer SS. The R_b rate was determined through nonlinear least-squares fitting to the $R_{2,app}$ data at various concentrations of DNA using Eq. 4 in the main text together with Eq. S2. In this fitting, R_b was the only fitting parameter. The calculation was performed with MATLAB.

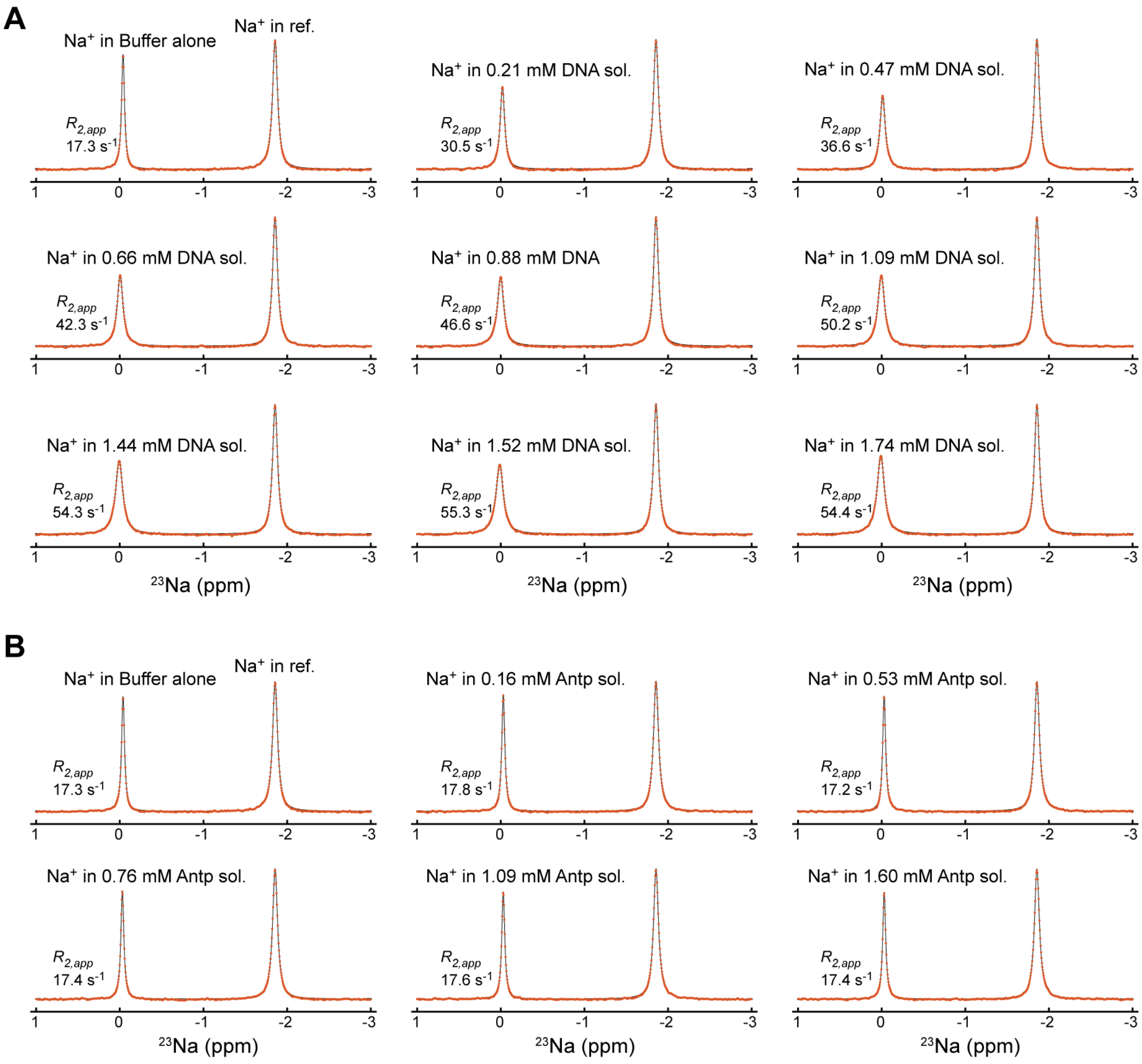

Fig. S2. NMR line shape data for spectra recorded through ^{23}Na BPP-LED experiments. Through the nonlinear least-squares fitting with a Lorentzian line-shape function (Eq. S1; n = 2), the apparent ^{23}Na transverse relaxation rates ($R_{2,app}$) were determined for Na^+ ions in buffer SS alone, in the solutions of the 15-bp DNA duplex (Panel A), and in the solutions of the Antp homeodomain (Panel B). Experimental data points from the ^{23}Na BPP-LED experiments are shown in red and the fitting results are shown in blue lines. Due to different degrees of decays during the ^{23}Na BPP-LED scheme, signal integrals do not directly represent [Na^+] for these datasets. The NMR data used for the line-shape analysis were identical to those used for Figure 2C-D in the main text. The determined ^{23}Na $R_{2,app}$ rates are plotted in Figure 4 in the main text.

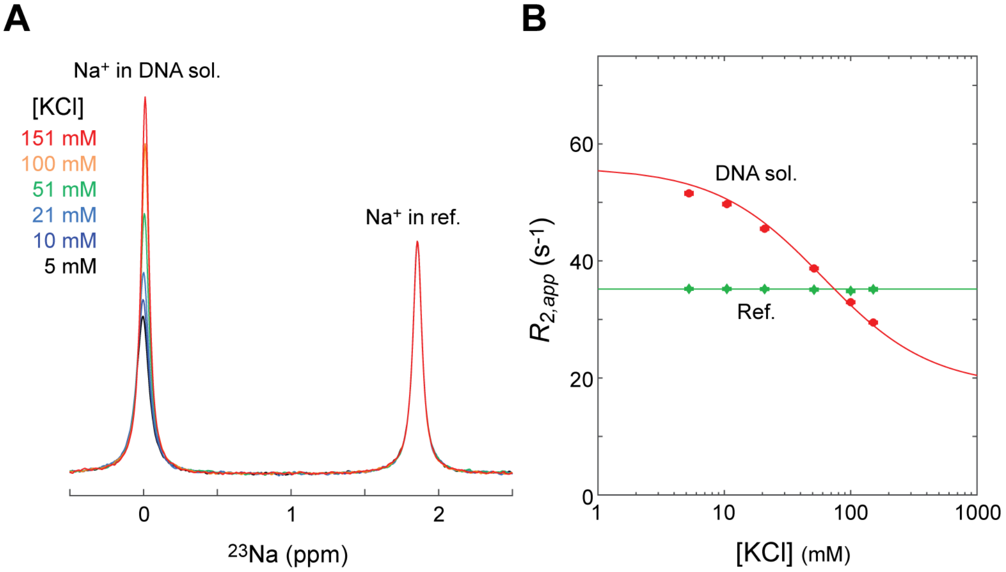

Fig. S3. Apparent ^{23}Na transverse relaxation rates measured for Na^+ ions in the DNA solutions at various concentrations of KCl. The data were obtained through the line-shape analysis of the ^{23}Na BPP-LED spectra, which were recorded to measure ^{23}Na diffusion for analysis of ionic competition between Na^+ and K^+ ions for the 15-bp DNA duplex (Figure 3A in the main text). (**A**) ^{23}Na spectra recorded through the ^{23}Na BPP-LED experiments. (**B**) Apparent ^{23}Na transverse relaxation rates determined from the line-shape analysis. The solid line represents the best-fit curve using Eq. 6 in which the diffusion coefficients D_b and D_f are replaced with the relaxation rates R_b (= 73.0 s^{-1}) and R_f (= 18.3 s^{-1}), respectively. The equation is equivalent to Eq. 5 of Bleam et al. (Ref. 37 in the main text). The competition parameter was determined to be 0.90.
